# The Relationship Between Problematic Internet Use, WhatsApp and Personality

**DOI:** 10.5964/ejop.2051

**Published:** 2022-02-25

**Authors:** Cristina Bernal-Ruiz, Ana Isabel Rosa-Alcázar

**Affiliations:** 1 Department of Personality, Assessment and Psychological Treatment, University of Murcia, Murcia, Spain; University of South Wales, Treforest, Wales, United Kingdom

**Keywords:** problematic internet use, WhatsApp, instant messaging, impulsivity, personality

## Abstract

The manner and frequency of Internet use may reflect the needs, preferences, values, personal motivations and personality characteristics of users. The aim of this research was to analyse the relationship between the Big Five Personality factors and impulsivity with Problematic Internet Use and the Negative Impact of WhatsApp. The sample consisted of 630 university students (75.7% female) aged between 18 and 62 years (M = 21.23). The results indicated that the Big Five Personality factors and impulsivity correlated significantly with Problematic Internet Use and the Negative Impact of WhatsApp. Impulsivity proved to be the most important predictor of Problematic Internet Use and the Negative Impact of WhatsApp. It can be concluded, cautiously, that impulsive people have a greater predisposition to develop Problematic Internet Use and, also to experience a greater negative impact due to the use of WhatsApp.

Internet access via mobile phone has increased considerably in recent years (Kim, Nam, Oh, & Kang, 2016). Despite the advantages that the use of smartphones and Internet access can afford, excessive use can lead to problematic use with difficulties similar to behavioural addictions (López-Fernandez, 2015).

## Problematic Internet Use and WhatsApp

Problematic Internet Use is growing phenomenon in our society. Despite increasing research in this field, there is no agreement today on a unified definition or criteria for its diagnosis (Acier & Kern, 2011; Billieux, 2012; Melchers, Li, Chen, Zhang, & Montag, 2015; Spada, 2014), which makes scientific understanding of this phenomenon difficult (Aboujaoude, 2010).

Among various online activities, it has been found that text messaging and instant messaging have been linked to Problematic Internet Use and mobile phone abuse (Buckner, Castille, & Sheets, 2012; Carbonell et al., 2012; Koo, 2010).

Instant messaging has several different features compared to other Internet applications that make this one more attractive to users. Features such as providing information about users' connection status, pop-ups when users receive a message, the immediacy of responses or the ability to have multiple simultaneous conversations -individually or in groups- (Mesch, Talmud, & Quan-Haase, 2012; Wang, Ngai, & Wei, 2012). WhatsApp is one of the most popular and widely used instant messaging applications for smartphones (Aharony & Gazit, 2016; Faye, Gawande, Tadke, Kirpekar, & Bhave, 2016; Montag et al., 2015). With total of 1,200 million active users in January 2017 (Statista, 2017), it is the leading instant messaging service in 109 countries, including Brazil, India, Mexico, Argentina, Russia, Germany, Spain and the United Kingdom (Schwartz, 2016). WhatsApp is a multi-platform instant messaging application for smartphones that allows: Message your friends and family for free (using your phone's Internet connection); Share messages, photos, and videos; Keep in touch with the groups of people that matter the most, like your family or coworkers; Talk to your friends or family for free (voice calls) or have face-to-face conversations through video calls (WhatsApp, 2019).

Not all Internet and/or WhatsApp users develop problematic use of the Internet/WhatsApp, there are certain variables that increase the vulnerability of individuals when experiencing such problematic use.

## Problematic Internet Use, WhatsApp and Personality

The way and frequency of Internet use may reflect the needs, preferences, values, personal motivations and personality characteristics of users (Zamani, Abedini, & Kheradmand, 2011). According to the model for the initiation and maintenance of psychological addiction (Echeburúa, 2000), there are personality traits that make some users more vulnerable to addiction than others. Specifically, a direct link has been found between neuroticism and Problematic Internet Use (Mark & Ganzach, 2014; Wang, Ho, Chan, & Tse, 2015). People with high neuroticism may use the Internet to seek information, express their emotions and experiences, or read others’ stories (Mark & Ganzach, 2014; Tan & Yang, 2014). Other studies associate low extraversion scores with Problematic Internet Use (Floros & Siomos, 2014; Servidio, 2014). According to Marriott and Buchanan (2014), introverts tended to be shyer in social situations and may be more comfortable interacting online rather than face-to-face.

Similarly, some research has found that conscientiousness, openness to experience and agreeableness are negatively related to Problematic Internet Use (Haddadain, Abedin, & Monirpoor, 2010; Kayiş et al., 2016; Müller et al., 2013; Puerta-Cortés, 2014; Samarein et al., 2013).

Impulsivity is another characteristic that has been associated with problematic Internet and smartphone use, increasing psychological vulnerability to addictions and being a risk factor for the development of Internet addiction (Beard, 2011; Cía, 2013; Koo & Kwon, 2014) and problematic use of mobile phones (Hwang & Park, 2015). People can use instant messaging to find immediate gratification when they are unable to find it in their social context (Roberts & Pirog, 2013). This may be one of the reasons why text messaging and instant messaging have been linked to Problematic Internet Use and mobile phone abuse (Buckner et al., 2012; Carbonell et al., 2012; Koo, 2010). Some authors report that the use of instant messaging is also positively related to openness to experience (Correa, Hinsley, & Gil de Zúñiga, 2010) and impulsiveness (Roberts & Pirog, 2013), and negatively to conscientiousness (Buckner et al., 2012) and neuroticism (Amiel & Sargent, 2004). Elsewhere, it has been reported that the need to control information leads neurotic people to a greater use of instant messaging (Ehrenberg, Juckes, White, & Walsh, 2008).

The present study aimed to analyse the relationship between the Big Five Personality Factors and Impulsivity with Problematic Internet Use and the Negative Impact of WhatsApp. The main novelties of the study are twofold: To jointly analyse Problematic Internet Use and WhatsApp and to evaluate the negative impact experienced by WhatsApp users through an innovative scale that has proven to have good psychometric properties and to be very useful (Bernal-Ruiz, Rosa-Alcázar, & González-Calatayud, 2019).

As a hypothesis we hope to contrast: 1) Problematic Internet Use will be directly related to Openness to experience and Neuroticism and inversely to Agreeableness, Extraversion and Conscientiousness. 2) WhatsApp's Negative Impact will be directly related to Openness to experience and Neuroticism and inversely to Agreeableness, Extraversion and Conscientiousness. 3) Impulsivity will be directly related to Problematic Internet Use and the Negative Impact of WhatsApp. 4) Low levels of Agreeableness, Conscientiousness, Neuroticism, Openness to experience, Extraversion, and Impulsivity will predict Problematic Internet Use. 5) Low levels of Agreeableness, Conscientiousness, Neuroticism, Openness to experience, Extraversion, and Impulsivity will predict the Negative Impact of WhatsApp.

## Method

### Participants

The sampling method used was for convenience. The sample consisted of 630 Spanish university students (75.7% women), aged between 18 and 62 years (*M* = 21.23 and *SD* = 4.32). All participants (100%) reported connecting to the Internet. Instant messaging was used by 100% of students. In addition, 100% used WhatsApp and 98.5% reported that WhatsApp was the most widely used of all instant messaging applications.

### Procedure

Teachers who taught at the University of Murcia and the Catholic University of Murcia were contacted by email or telephone to request their participation in the study. A briefing meeting was held with each of the teachers who showed interest in collaborating.

The university students, who agreed to participate and signed the consent form, completed an anonymous questionnaire in a 1-hour session in the presence of a professor and a member of the research team (clinical evaluation expert). The students received no incentives whatsoever for participating in the study. The research was evaluated and accepted by the Ethics Commission of the University of Murcia (Spain), so guaranteeing it respected the ethical principles of research with human beings.

### Instruments

#### Generalized Problematic Internet Use Scale

The Generalized Problematic Internet Use Scale (GPIUS2, Caplan, 2010; Gámez-Guadix, Orue, & Calvete, 2013) evaluates widespread Problematic Internet Use. It consists of 15 Likert-type formatted items with values ranging from 1 (strongly disagree) to 6 (strongly agree). The scale measures five dimensions: 1) Preference for online social Interactions, α = 0.90, 2) Mood regulation, α = 0.78, 3) Cognitive preoccupations, α = 0.76, 4) Compulsive Internet use, α = 0.84, and 5) Negative outcomes, α = 0.79. All reliability coefficients were calculated in the sample used in this study. For full scale, the alpha coefficient was 0.91.

#### WhatsApp Negative Impact Scale

The WhatsApp Negative Impact Scale (WANIS; Bernal-Ruiz et al., 2019) evaluates the main problems that can arise from the excessive use of this application. It comprises 37 Likert-type format items with values ranging from 0 (Strongly disagree) to 3 (Strongly agree). It presents a trifactorial structure: 1) Negative Consequences of WhatsApp use, α = 0.93, 2) Controlling Intimate Relationships through WhatsApp, α = 0.85, and 3) Problematic Use of WhatsApp, α = 0.87. For the full scale, the alpha coefficient was 0.95.

#### Reduced NEO Five-Factor Inventory

The Reduced NEO Five-Factor Inventory (NEO-FFI; Costa & McCrae, 1992) is a reduced version of the Neo-Revised Personality Inventory, which allows a quick and general measurement of the five personality factors. It is composed of 60 Likert-type items with 5 answer choices ranging from 0 (total disagreement) to 4 (total agreement). The items are grouped in 5 dimensions: 1) Neuroticism, α = 0.82, 2) Extraversion, α = 0.84, 3) Openness to Experience, α = 0.77, 4) Agreeableness, α = 0.71 and 5) Conscientiousness, α = 0.82. For the full scale the alpha coefficient was 0.78.

#### State Impulsivity Scale

The State Impulsivity Scale (SIS; Iribarren, Jimenez-Gimenez, Garcia-de Cecilia, & Rubio-Valladolid, 2011) evaluates impulsivity as short-term variable behaviour. It consists of 20 Likert-type format items with values ranging from 0 (almost never) to 3 (almost always/always). The items are distributed in three dimensions: 1) Reward α = 0.85, 2) Automatism, α = 0.82, and 3) Attentional, α = 0.86. For the full scale, the alpha coefficient was 0.93.

### Data Analysis

Data analysis was performed using the SPSS statistical package (v. 22.0). *t*-tests were calculated for independent samples. Pearson correlations and stepwise linear regression analyses were conducted to examine the predictive ability of the Big Five personality factors and impulsivity in Problematic Internet Use and the Negative Impact of WhatsApp.

## Results

### Influence of Sex and Age on Problematic Internet Use and Negative Impact of WhatsApp

In relation to sex, statistically significant differences were found in the problematic use of WhatsApp, *t*(627) = 2.722, *p* = .007, and in the total WhatsApp Negative Impact Scale score, *t*(627) = 2.184, *p* = .029, with women obtaining the highest scores (Table 1).

**Table 1:** Comparison of Means on the Generalized Problematic Internet Use Scale (GPIUS) and the WhatsApp Negative Impact Scale (WANIS) Based on Sex (N = 630)

Variable/Sex	*n*	*M*	*SD*	*t* (*gl*)	*p*
POSI					
Female	476	5.61	3.19	−0.147 (627)	.142
Male	153	6.04	3.08		
MR					
Female	476	9.77	3.84	0.463 (284,188)	.644
Male	153	9.62	3.43		
CP					
Female	476	7.72	3.43	−0.032 (290,356)	.974
Male	153	7.73	3.00		
CIU					
Female	476	8.09	3.83	−0.214 (305,939)	.831
Male	153	8.16	3.18		
NO					
Female	476	5.93	3.28	−1.258 (627)	.209
Male	153	6.31	3.01		
GPIUS (G)					
Female	476	37.12	13.69	−0.591 (627)	.554
Male	153	37.86	12.12		
NCWU					
Female	476	12.29	9.07	1.388 (627)	.166
Male	153	11.12	9.06		
CIRW					
Female	476	11.75	6.38	1.706 (627)	.088
Male	153	10.74	6.31		
WPU					
Female	476	17.95	7.04	2.722 (627)	**.007**
Male	153	16.14	7.38		
WANIS					
Female	476	41.99	19.50	2.184 (627)	**.029**
Male	153	38.01	19.94		

*Note*. *SD* = standard deviation; POSI = Preference for Online Social Interaction; MR = Mood Regulation; CP = Cognitive Preoccupation; CIU = Compulsive Internet Use; NO = Negative Outcomes; GPIUS (G) = Overall Score on the Generalized Problematic Internet Use Scale; NCWU = Negative Consequences of WhatsApp Use; CIRW = Controlling Intimate Relationships through WhatsApp; WPU = WhatsApp's problematic use; WANIS = WhatsApp's Negative Impact Scale total score; Bold = statistical significance.

Age had very low correlations with both the Problematic Internet Use *r* = −.12, *p* < .01, and the Negative Impact of WhatsApp, *r* = −.13, *p* < .01.

### The Big Five Personality Factors and Problematic Internet Use

The highest correlation was observed between Extraversion and Preference for Online Social Interaction, *r* = −.47, *p* < .01. The GPIUS total score correlated significantly with all personality dimensions (Table 2), as well as with the total score in the Reduced NEO Five-Factor Inventory, returning the highest correlations with Agreeableness *r* = −.35, *p* < .01 and Conscientiousness, *r* = −.34, *p* < .01.

**Table 2:** Pearson’s Correlations Between the Generalized Problematic Internet Use Scale (GPIUS) and the NEO Five-Factor Inventory (NEO-FFI) (N = 630)

Variable	*M*	*SD*	1	2	3	4	5	6	7	8	9	10	11
**1. POSI**	5.71	3.16											
**2. MR**	9.74	3.74	.36**										
**3. CP**	7.74	3.34	.43**	.49**									
**4. CIU**	8.12	3.69	.38**	.41**	.78**								
**5. NO**	6.02	3.22	.57**	.35**	.63**	.65**							
**6. GPIUS (G)**	37.34	13.33	.69**	.69**	.86**	.83**	.81**						
**7. NEU**	23.20	7.84	.19**	.23**	.20**	.23**	.24**	.28**					
**8. EXT**	30.77	7.40	−.47**	*ns*	*ns*	−.10*	−.21**	−.22**	−.27**				
**9. OPE**	29.18	7.53	−.21**	*ns*	−.21**	−.18**	−.17**	−.19**	*Ns*	.25**			
**10. AGR**	28.53	6.21	−.33**	−.17**	−.26**	−.24**	−.36**	−.35**	−.26**	.20**	.17**		
**11. CON**	29.62	6.94	−.29**	−.14**	−.22**	−.31**	−.37**	−.34**	−.25**	.26**	.16**	.36**	
**12. NEO-FFI**	141.31	17.88	−.43**	*ns*	−.20**	−.22**	−.33**	−.30**	.17**	.57**	.67**	.53**	.58**

*Note. SD* = standard deviation; POSI = Preference for Online Social Interaction; MR = Mood Regulation; CP = Cognitive Preoccupation; CIU = Compulsive Internet Use; NO = Negative Outcomes; GPIUS (G) = Overall Score on the Generalized Problematic Internet Use Scale; NEU = Neuroticism; EXT = Extraversion; OPE = Openness to experience; AGR = Agreeableness; CON = Conscientiousness; NEO-FFI = NEO Five-Factor Inventory; ns = no significant correlation.

*p < .05. **p < .01.

### The Big Five Personality Factors and the Negative Impact of WhatsApp

All the correlations between the Big Five and the WANIS scale factors were significant except for the correlation between Extraversion and Problematic Use of WhatsApp (Table 3). Most of the significant correlations were negative except in the case of Neuroticism where, as with Problematic Internet Use, the correlations were positive. The highest correlations were observed between Negative Consequences of WhatsApp Use and Conscientiousness, *r* = −.41, *p* < .01 and Negative Consequences and Agreeableness, *r* = −.37, *p* < .01. As for the total WANIS score, it correlated significantly with all personality dimensions, as well as with the total score in the Reduced NEO Five-Factor Inventory, achieving the highest correlations with Conscientiousness, *r* = −.37, *p* < .01 and Agreeableness, *r* = −.36, *p* < .01.

**Table 3:** Pearson’s Correlations Between the WhatsApp Negative Impact Scale (WANIS) and the NEO Five-Factor Inventory (NEO-FFI) (N = 630)

Variable	*M*	*SD*	1	2	3	4	5	6	7	8	9
**1. NCWU**	12.01	9.07									
**2. CIRW**	11.52	6.38	.67**								
**3. WPU**	17.53	7.17	.64**	.57**							
**4. WANIS**	41.06	19.68	.91**	.84**	.84**						
**5. NEU**	23.20	7.84	.24**	.25**	.28**	.29**					
**6. EXT**	30.77	7.40	−.22**	−.12**	*Ns*	−.15**	−.27**				
**7. OPE**	29.18	7.53	−.26**	−.24**	−.21**	−.27**	*ns*	.25**			
**8. AGR**	28.53	6.21	−.37**	−.25**	−.28**	−.36**	−.26**	.20**	.17**		
**9. CON**	29.62	6.94	−.41**	−.24**	−.28**	−.37**	−.25**	.26**	.16**	.36**	
**10. NEO-FFI**	141.31	17.88	−.38**	−.22**	−.18**	−.31**	.17**	.57**	.67**	.53**	.58**

*Note. SD* = standard deviation; NCWU = Negative Consequences of WhatsApp Use; CIRW = Controlling Intimate Relationships through WhatsApp; WPU = WhatsApp's problematic use; WANIS = WhatsApp’s Negative Impact Scale total score; NEU = Neuroticism; EXT = Extraversion; OPE = Openness to experience; AGR = Agreeableness; CON = Conscientiousness; NEO-FFI = NEO Five-Factor Inventory; ns = no significant correlation.

**p < .01.

### Impulsivity and Problematic Internet Use

All the components of the State Impulsivity Scale (SIS)—Reward, Automatism and Attentional—correlated significantly and directly with the variables of Problematic Internet Use (Table 4). The correlations between Reward and each of the GPIUS factors stood out, with the highest being with Negative Outcomes, *r* = .58, *p* < .01. Negative Outcomes was the dimension of the GPIUS that obtained higher correlations with all the components of Impulsivity. The GPIUS total score correlated with the total SIS score and with all dimensions of Impulsivity, and the highest correlation was with Reward, *r* = .58, *p* < .01.

**Table 4:** Pearson’s Correlations Between the Generalized Problematic Internet Use Scale (GPIUS) and the State Impulsivity Scale (SIS) (N = 630)

Variable	*M*	*SD*	1	2	3	4	5	6	7	8	9
**1. POSI**	5.71	3.16									
**2. MR**	9.74	3.74	.36**								
**3. CP**	7.74	3.34	.43**	.49**							
**4. CIU**	8.12	3.69	.38**	.41**	.78**						
**5. NO**	6.02	3.22	.57**	.35**	.63**	.65**					
**6. GPIUS (G)**	37.34	13.33	.69**	.69**	.86**	.83**	.81**				
**7. REW**	6.14	4.20	.45**	.30**	.47**	.47**	.58**	.58**			
**8. AUT**	5.13	3.65	.41**	.24**	.40**	.41**	.48**	.50**	.68**		
**9. ATT**	7.01	4.42	.35**	.20**	.40**	.42**	.48**	.47**	.65**	.74**	
**10. SIS**	18.29	10.94	.45**	.28**	.47**	.49**	.57**	.58**	.88**	.90**	.90**

*Note. SD* = standard deviation; POSI = Preference for Online Social Interaction; MR = Mood Regulation; CP = Cognitive Preoccupation; CIU = Compulsive Internet Use; NO = Negative Outcomes; GPIUS (G) = Overall Score on the Generalized Problematic Internet Use Scale; REW = Reward; AUT = Automatism; ATT = Attentional; SIS = State Impulsivity Scale.

**p < .01.

### Impulsivity and Negative Impact of WhatsApp

The correlations of Impulsivity with the Negative Impact of WhatsApp were significant and direct (Table 5). Reward was the dimension that had the highest significant correlations with each of the factors on the WANIS scale. As for the Negative Impact of WhatsApp, Negative Consequences was the factor that reached the highest significant correlations with the components of Impulsivity. The WANIS total score correlated significantly with all dimensions of the SIS as well as with the total score on this scale, *r* = .58, *p* < .01.

**Table 5:** Pearson’s Correlations Between the WhatsApp Negative Impact Scale (WANIS) and the State Impulsivity Scale (SIS) (N = 630)

Variable	*M*	*SD*	1	2	3	4	5	6	7
**1. NCWU**	12.01	9.07							
**2. CIRW**	11.52	6.38	.67**						
**3. WPU**	17.53	7.17	.64**	.57**					
**4. WANIS**	41.06	19.68	.91**	.84**	.84**				
**5. REW**	6.14	4.20	.55**	.41**	.44**	.55**			
**6. AUT**	5.13	3.65	.48**	.38**	.43**	.51**	.68**		
**7. ATT**	7.01	4.42	.50**	.37**	.43**	.51**	.65**	.74**	
**8. SIS**	18.29	10.94	.58**	.43**	.49**	.58**	.88**	.90**	.90**

*Note. SD* = standard deviation; NCWU = Negative Consequences of WhatsApp Use; CIRW = Controlling Intimate Relationships through WhatsApp; WPU = WhatsApp's problematic use; WANIS = WhatsApp’s Negative Impact Scale total score; REW = Reward, AUT = Automatism; ATT = Attentional; SIS = State Impulsivity Scale.

**p < .01.

### Predictive Variables of Problematic Internet Use and Negative Impact of WhatsApp

To examine the predictive power of the Big Five Personality Factors and Impulsivity on Problematic Internet Use, a multiple linear stepwise regression analysis was performed (Table 6).

**Table 6:** Stepwise Regression Results for Generalized Problematic Internet Use Scale (GPIUS)

GPIUS variable / Predictor	ß	*t*	Adjusted *R*^2^
POSI			
Extraversion	−0.427	−13.001***	.384
Impulsivity	0.385	10.202***	
Neuroticism	−0.082	−2.361*	
Agreeableness	−0.077	−2.100*	
MR			
Impulsivity	0.225	5.535***	.094
Neuroticism	0.149	3.665***	
CP			
Impulsivity	0.449	12.480***	.231
Openness to experience	−0.100	−2.775**	
CIU			
Impulsivity	0.443	11.185***	.246
Conscientiousness	−0.098	−2.460*	
NO			
Impulsivity	0.514	13.953***	.350
Extraversion	−0.119	−3.577***	
Conscientiousness	−0.089	−2.357*	
GPIUS (G)			
Impulsivity	0.560	17.265***	.352
Extraversion	−0.136	−4.203***	

*Note.* POSI = Preference for Online Social Interaction; MR = Mood Regulation; CP = Cognitive Preoccupation; CIU = Compulsive Internet Use; NO = Negative Outcomes; GPIUS (G) = Overall Score on the Generalized Problematic Internet Use Scale.

*p < .05. **p < .01. ***p < .001.

Problematic Internet Use was explained by Impulsivity and Extraversion (35.2%). The most widely explained variable of the pathological use of the Internet was Preference for Online Social Interaction (38.4%), with four predictors: Extraversion, Impulsivity, Neuroticism and Agreeableness.

Impulsivity was the greatest predictor in all cases except in the Preference for Online Social Interaction, where it was Extraversion (β = −0.427).

The Negative Impact of WhatsApp was explained by Impulsivity, Openness to Experience, Neuroticism and Conscientiousness (37.6%). The most explained variable of the Negative Impact of WhatsApp was Negative Consequences of WhatsApp Use (37.3%), with four predictors: Impulsivity, Conscientiousness, Openness to Experience and Extraversion (Table 7).

**Table 7:** Stepwise Regression Results for WhatsApp Negative Impact Scale (WANIS)

WANIS variable / Predictor	ß	*t*	Adjusted *R*^2^
NCWU			
Impulsivity	0.470	12.774***	.373
Conscientiousness	−0.142	−3.839***	
Openness to experience	−0.101	−3.015**	
Extraversion	−0.094	−2.818**	
CIRW			
Impulsivity	0.348	8.847***	.221
Openness to experience	−0.166	−4.516***	
Neuroticism	0.132	3.464**	
WPU			
Impulsivity	0.402	10.576***	.271
Neuroticism	0.177	4.595***	
Openness to experience	−0.155	−4.191***	
Extraversion	0.119	3.236**	
WANIS			
Impulsivity	0.461	11.941***	.376
Openness to experience	−0.156	−4.754***	
Neuroticism	0.114	3.334**	
Conscientiousness	−0.090	−2.492*	

*Note.* NCWU = Negative Consequences of WhatsApp Use; CIRW = Controlling Intimate Relationships through WhatsApp; WPU = WhatsApp's problematic use; WANIS = WhatsApp’s Negative Impact Scale total score;1.

*p < .05. **p < .01. ***p < .001.

Impulsivity was a variable predictor of the Negative Impact of WhatsApp and each of its component dimensions. It was also the strongest predictor in all cases. Openness to Experience was another personality trait that predicted both the Negative Impact of WhatsApp and all its dimensions.

## Discussion and Conclusion

The first objective was to examine the influence of sociodemographic variables on Problematic Internet Use and on the Negative Impact of WhatsApp. No statistically significant differences were observed in Problematic Internet Use by sex (Sebena, Orosova, & Benka, 2013; Wang et al., 2011). In relation to WhatsApp, statistically significant differences were observed in the problematic use of WhatsApp and in the total score of the WANIS, with the highest average scores corresponding to women in both cases (Montag et al., 2015). Women use WhatsApp for longer periods of time (Faye et al., 2016).

Regarding age, our results support previous studies that considered adolescents and young people to be the most vulnerable users for the development of Problematic Internet Use (Lam, 2014; Wu, Lee, Liao, & Chang, 2015). Compared to older students, younger university students may experience more emotional instability and crisis, and may use the Internet as a means of helping to alleviate problems experienced (Koo & Kwon, 2014).

Age correlated significantly and negatively with the Negative Impact of WhatsApp and all factors of the WANIS scale. Therefore, the younger the age of the university students, the more negative consequences that were derived from its use, the greater control of intimate relationships made through WhatsApp and the more problematic the use that was experienced. This may be because young people are more vulnerable to addiction in general and to Problematic Internet Use in particular (Lam, 2014; Wu et al., 2015), possibly because they have less self-control, less self-regulation and are more exposed to the Internet and new technologies. In addition, young people are less aware of the negative consequences of their actions, which would lead them to continue with addictive behaviours and experience an increasing negative impact. Finally, relationships between young couples are often less stable and shorter-lasting. This, combined with the insecurity that characterises the younger, would lead to the need for greater control of the partner, with WhatsApp being a tool that could be used for this purpose.

The second objective was to analyse the relationship between problematic Internet use and the Negative Impact of WhatsApp on the Big Five personality factors.

Problematic Internet use correlated significantly and negatively with Agreeableness, Conscientiousness, Openness to experience and Extraversion and directly with Neuroticism. Our results are consistent with studies that found a negative relationship between Problematic Internet Use and Agreeableness (Floros & Siomos, 2014; Kayiş et al., 2016), Conscientiousness (Samarein et al., 2013; Wang et al., 2015), Openness to Experience (Haddadain et al., 2010; Kayiş et al., 2016) and Extraversion (Floros & Siomos, 2014). It is also in line with studies that found a direct link between Problematic Internet Use and neuroticism (Mark & Ganzach, 2014; Wang et al., 2015). These results only partially confirm Hypothesis 1, which stated that Problematic Internet Use would be directly related to Openness to experience, Neuroticism and Impulsivity and inversely to Agreeableness, Extraversion and Conscientiousness.

Problematic Internet Use had the highest correlations with Agreeableness and Conscientiousness. People with low agreeableness are seen by others as less pleasant, have difficulty establishing peer networks, and participate less frequently in group activities. These people may be rejected by others, leading them to turn to the Internet as a means of fulfilling their personal needs (Samarein et al., 2013).

Individuals with low conscientiousness may be at greater risk of being distracted by all the possibilities offered by the Internet and of getting lost in virtual worlds (Müller et al., 2013). Due to the difficulties in organization, self-discipline, compliance with rules and commitments that characterize them (Puerta-Cortés, 2014), they may prefer to connect to the Internet instead of engaging in other less pleasant activities, with the consequent risk of developing Problematic Internet Use.

Negative Impact of WhatsApp correlated significantly and negatively with Agreeableness, Conscientiousness, Openness to experience and Extraversion, and directly with Neuroticism. These results partially confirm Hypothesis 2 which formulated that the Negative Impact of WhatsApp will be directly related to Openness to experience and Neuroticism and inversely to Agreeableness, Extraversion and Conscientiousness.

These results are consistent with previous studies linking instant messaging to conscientiousness (Buckner et al., 2012) and neuroticism (Ehrenberg et al., 2008). Decreased conscientiousness was the personality trait that was most strongly related to the increased negative consequences experienced by using WhatsApp. Thus, people with low conscientiousness may find in WhatsApp an application to spend time, talk to others and entertain themselves, leaving aside or postponing their daily activities, which could lead to long-term problems in their daily functioning, academic performance, family and social relationships.

The third objective was to analyse the relationship between the Problematic Internet Use and Negative Impact of WhatsApp with Impulsivity. The results indicated that Impulsivity was directly associated with Problematic Internet Use and the Negative Impact of WhatsApp, Hypothesis 3 being confirmed. This is congruent with previous research (Beard, 2011; Cía, 2013; Echeburúa & Requesens, 2012; Koo & Kwon, 2014). Impulsivity has not only been considered a risk factor for the development of Problematic Internet Use (Echeburúa & Requesens, 2012), but it has also been proven that like behavioural addiction progresses, behaviours become automatic and are activated by impulses and emotions over which one has poor cognitive and self-critical control (Cía, 2013).

Reward was the dimension of Impulsivity that obtained the highest significant correlations with each of the factors of Problematic Internet Use scale and the WANIS. We would highlight that Reward correlations with Negative Outcomes and with Negative Consequences of WhatsApp use. People with Problematic Internet Use may turn to the Internet to satisfy the need for immediate gratification with no regard to the possible negative consequences of this behaviour (Cía, 2013). Similarly, users of WhatsApp would seek immediate rewards through this application without taking into consideration the negative consequences that may result from their behaviour (Cía, 2013).

The final objective was to examine the predictive power of the Big Five personality factors and Impulsivity on Problematic Internet Use and Negative Impact of WhatsApp.

According to our results, the variables that predict Problematic Internet Use were Impulsivity and Extraversion. These results only partially confirm Hypothesis 4 which postulated that low levels of agreeableness, conscientiousness, neuroticism, openness to experience, extraversion and Impulsivity would predict Problematic Internet Use. From these results, we would indicate, although with caution, that impulsive and not very extrovert people could have a greater predisposition to develop Problematic Internet Use. Impulsive and introverted people may have an immediate need to interact with others but may not be able to relate to them because of their shyness. These people may impulsively turn to the Internet to interact with others in a more comfortable and secure way and thus immediately achieve the social gratification they cannot gain from face-to-face interactions (Marriott & Buchanan, 2014).

The variables that predicted the Negative Impact of WhatsApp were Impulsivity, Openness to Experience, Neuroticism and Conscientiousness, therefore Hypothesis 5 is only partially confirmed. We can say, albeit cautiously, that people who are impulsive, neurotic, less inclined toward change or novelty, and less conscientious may be more likely to experience a negative impact because of their use of WhatsApp. These people could find in WhatsApp a way of keeping in touch with friends and acquaintances in a comfortable, safe and familiar way.

Our results seem to indicate that Impulsivity plays a relevant role in the appearance and maintenance of both problems. At first, impulsive people might turn to the Internet or WhatsApp to immediately satisfy a particular need or desire. Over time, it may be that these people will cease to use WhatsApp or the Internet in a controlled and voluntary way and move towards compulsive or recurrent use, because they are unable to suppress their impulses. This could encourage the development of problematic Internet and/or WhatsApp use and lead them to experience a series of negative consequences similar to those of behavioural addictions (López-Fernandez, 2015).

This study has afforded a deeper understanding of problematic Internet and WhatsApp use, two phenomena that, although recent, are increasingly present in our society. This research provides evidence about the relationship of these two issues to the Big Five personality factors and Impulsivity. Specifically, the importance of Impulsivity in problematic Internet and WhatsApp use is clear because, according to the results, impulsive people may have a greater predisposition to develop both problems. This finding is essential for both the prevention and treatment of problematic Internet and WhatsApp use. Hence, programs or workshops that teach how to control and manage impulsivity, train skills to resist the temptation of an immediate reward, learn to expect a later reward, etc. could be an effective way to reduce the risk of the emergence of Problematic Internet Use and decrease the negative impact experienced by using WhatsApp.

As limitations of the study we can highlight the use of a sample for convenience, which influences the generalization of the results. On the other hand, it is a transversal study, so preventing the establishment of causal relationships that would better explain the direction of the variables used.
